# Two-color spheroid model for determining the O_2_-induced radiosensitivity of HNSCC

**DOI:** 10.1186/s13036-025-00611-y

**Published:** 2026-01-08

**Authors:** Danny Knobloch-Sperlich, Matthias Kappler, Markus Glaß, Antje Güttler, Marina Petrenko, Jonas Pyko, Tony Gutschner, Frank Tavasol, Dirk Vordermark, Matthias Bache

**Affiliations:** 1 Department of Radiotherapy, Martin Luther University Halle-Wittenberg, Ernst-Grube- Straße 40, Halle (Saale), Germany; 2 Department of Oral and Maxillofacial Plastic Surgery, Martin Luther University Halle- Wittenberg, Ernst-Grube-Straße 40, Halle (Saale), Germany; 3 Institute of Molecular Medicine, Section for Molecular Cell Biology, Faculty of Medicine, Martin Luther University Halle-Wittenberg, Halle (Saale), Germany; 4 Institute of Molecular Medicine, Section for RNA Biology and Pathogenesis, Faculty of Medicine, Martin Luther University Halle-Wittenberg, Halle (Saale), Germany

**Keywords:** HNSCC, Normoxia, Hypoxia, 3D spheroid model, Radiosensitivity, Biological engineering, Tumor microenvironment, Ascorbic acid, Oxygen enhancement ratio (OER)

## Abstract

Hypoxia strongly affects the growth, invasion, and therapeutic response of solid tumors, including head and neck squamous cell carcinoma (HNSCC). Despite intensive research, only a few substances have progressed to clinical trials as radiosensitizers. Therefore, new clinically relevant tumor models are needed to identify agents that overcome radiation resistance in hypoxic tumors. To study radiosensitivity of hypoxic and normoxic cells, we developed a two-color spheroid model using two HNSCC cell lines, SAS and FaDu, with GFP-labeled inner cell layers and mCherry-labeled outer layers. Optimizing the ratios of fluorescent cells enabled formation of hypoxic and normoxic zones, confirmed by pimonidazole, HIF1α, and CA IX staining. A newly established fluorescence clonogenic survival assay demonstrated transferability of results from 2D normoxia and hypoxia assays to these 3D model. The inner GFP-labeled cells showed significantly lower plating efficiency and increased radiation resistance compared to outer mCherry-labeled cells, similar to 2D hypoxic cells. To improve the model by reducing normoxia-induced HIF1α expression in the outer layer, we added physiological concentrations of ascorbic acid. Ascorbic acid also increased spheroid growth, clonogenic survival, and radioresistance under normoxia, while hypoxic responses remained unchanged. These two-layer spheroid model with distinct fluorescent labels provide a simple, robust assay to distinguish hypoxic from normoxic tumor areas in radiotherapy research. Addition of ascorbic acid further refines the physiological relevance of 3D tumor models and modulates radiosensitivity of the outer mCherry-labeled cell layer in both HNSCC models.

**Supplementary Information:**

The online version contains supplementary material available at 10.1186/s13036-025-00611-y.

## Background

Head and neck squamous cell carcinoma (HNSCC) is the sixth most common cancer worldwide, with 890,000 new cases and 460,000 deaths in 2022 [1]. The major risk factors for HNSCC are exposure to tobacco smoke and alcohol consumption. For effective tumor treatment, the common therapeutic approaches are radical surgery, chemotherapy and irradiation. However, one characteristic of HNSCC is the presence of a hypoxic microenvironment in the inner tumor tissue [2–4]. Hypoxia is a negative prognostic factor and is associated with the stabilization of HIF1α, a key transcription factor that causes a switch in metabolic pathways and is associated with radioresistance [5, 6]. These findings confirm the critical role of hypoxia in the tumor microenvironment, which promotes tumor progression and causes therapy resistance. Therefore, there is an urgent need for improved diagnostic tools and research models to better understand the resistance mechanisms of HNSCC [7, 8].

Two-dimensional (2D) cell culture-generated tumor models are efficient and cost effective. However, 2D models are inadequate for replicating the structural and biochemical complexity of in vivo tumors [9–11]. To address the limitations of 2D models in studying the complex interplay between hypoxic and normoxic cells, three-dimensional (3D) tumor models have emerged as powerful tools [12, 13]. These 3D models can much better mimic the spatial organization and gradients of oxygen, nutrients, and metabolic waste products, which are found in solid tumors [14]. 3D models therefore enable a more accurate investigation of tumor biology, including cell–cell and cell–matrix interactions [2, 12, 15]. The radiobiological response is influenced by variations in proliferation, oxygen levels and cell viability [16]. Under hypoxia, tumor cells exhibit substantially reduced sensitivity to ionizing radiation. This is due to the absence of oxygen, which is a crucial amplifier of radiation-induced DNA damage [17]. The quantitative description of this phenomenon is facilitated by the oxygen enhancement ratio (OER), which is typically observed to range between two and three in numerous 2D tumor cell models, including those of HNSCC [18, 19]. The OER describes that, under hypoxic conditions, a two- to threefold higher radiation dose is required to elicit a biological response comparable to that under normoxic conditions [15, 18]. Various 3D tumor models have been established to study oxygen heterogeneity, including spheroid, reporter-based, and microfluidic systems that have substantially advanced the visualization and quantification of hypoxia for a better understanding of hypoxia dynamics [20–34]. However, a discrimination between spatially defined hypoxic and normoxic regions using the clonogenic survival test, the gold standard for determining radiation sensitivity, was technically not implemented [25, 35]. The present two-color spheroid model overcomes this limitation by enabling differential analysis of clonogenic survival within distinct oxygen compartments, thereby providing the basis for a functional evaluation of hypoxia-induced therapy resistance.

In order to achieve a more accurate 3D model, the micronutrient ascorbic acid facilitates the reproduction of a physiological tumor microenvironment. It plays an essential role in a variety of physiological processes, including its function as an antioxidant and a cofactor in enzymatic reactions at physiological concentrations [36]. Furthermore, ascorbic acid enables a clearer distinction between hypoxic and normoxic zones, because it is involved in the regulation of various cancer-related proteins, including HIF1α and prolyl 4-hydroxylase [37–39]. Moreover, researchers discovered that the cancers with the most potent HIF1 function were those lacking ascorbic acid in the tumor microenvironment [40, 41].

Here, we present a two-color spheroid model for HNSCC combined with a newly established fluorescence clonogenic assay, enabling the investigation of the response to irradiation in differently oxygenated layers within the spheroid, which could be refined by addition of ascorbic acid. This novel approach to distinguishing different zones in 3D tumor models could facilitate the evaluation of radiosensitizers being developed against therapy-resistance of hypoxic HNSCC.

## Methods

### Cell culture conditions and treatment of cells

The HNSCC cell lines SAS and FaDu were kindly received by Prof. Zips, Department of Radiation Oncology and Radiotherapy, University Hospital Charité Berlin, Germany. The cells were cultured at 37 °C, 20% O_2_ and 5% CO_2_ with RPMI 1640 medium (Thermo Fisher Scientific, Waltham, MA, USA) containing 10% fetal bovine serum (Capricorn Scientific, Ebsdorfergrund, Germany), 1% sodium pyruvate (Gibco, Thermo Fisher Scientific, Scotland, UK) and 2% penicillin/streptomycin (Sigma-Aldrich, St. Louis, MO, USA). It should be noted that 2% O_2_ in standard incubators corresponds to approximately 1% O_2_ at the cellular level. L-ascorbic acid (Sigma-Aldrich) was dissolved in distilled H_2_O to obtain a 20 mM stock solution, which was sterilely filtered. The cells were treated with 10 mg/l (0.06 mM) ascorbic acid for up to 24 h at 37 °C, depending on the assay performed. The Gas Pak EZ Anaerobic Pouch System (BD Biosciences, Heidelberg, Germany) was used to generate hypoxic conditions (approximately 0.1% O_2_). Irradiation was performed using a Synergy linear accelerator (Elekta, Stockholm, Sweden). For determination of radiosensitivity, the cells were irradiated with 6 MV photon radiation between 2 Gy and 16 Gy.

### Two-color spheroid formation

SAS and FaDu cells were subjected to lentiviral transfection using coding vectors containing the genes for green fluorescent protein (GFP, pLV-eGFP, Addgene plasmid 36083) and red fluorescent protein (mCherry pLV-mCherry, Addgene plasmid 36084, both gifts from Pantelis Tsoulfas) as described previously [42]. To analyze the zones with different oxygen conditions within the spheroids, two-layered spheroids consisting of these fluorescence-labeled SAS or FaDu cells were constructed. Briefly, on the first day, 10,000 GFP-labeled SAS or 8,000 GFP-labeled FaDu cells were seeded into polymeric coated 96-round-bottom-well plates (faCellitate, Mannheim, Germany) and centrifuged. The next day, 40,000 mCherry-labeled SAS or, after two days, 32,000 mCherry-labeled FaDu cells were seeded on top of the GFP-labeled cells. A ratio of 1:4 (GFP-labeled to mCherry-labeled cells) was necessary to form a complete outer layer. After 48 h, the SAS cells formed multilayered 3D spheroids, whereas the FaDu cells formed them after 72 h. Growth rates (diameter and density) were documented with a GelCount plate reader (Oxford Optronix, Adderbury, UK).

### 2D clonogenic survival and radiosensitivity

The procedure was conducted as described [43]. Briefly, cells were seeded into 6-well plates and incubated for 24 h prior to treatment with irradiation under normoxic and hypoxic conditions. Afterwards, the cells were trypsinized and counted, and 400 to 2,000 cells were seeded. After 10 to 14 days, the colonies were fixed with 3.7% paraformaldehyde (Chemsolute, Renningen, Germany) and stained with 10% Giemsa solution (Sigma‒Aldrich). The flasks were scanned and analyzed with a GelCount plate reader (Oxford Optronix). A minimum of 50 cells per colony were set to count as colonies. The ratio of the number of colonies formed after irradiation and treatment was compared to the number of colonies formed in nonirradiated controls. The cell survival curves were fitted to a linear quadratic model (− lnS = αD + βD^2^) via Origin 2019. The OER were calculated as the quotient of the radiation doses resulting in 10% survival of hypoxic/normoxic or treated cells.

### 3D clonogenic survival and radiosensitivity

The spheroids were irradiated 24 h after treatment with ascorbic acid. Depending on the cell line, different doses between 2 Gy and 16 Gy were used. For the clonogenic survival assay, irradiated spheroids were trypsinized, and 600 to 20,000 cells were seeded into cell culture flasks depending on the treatment and irradiation dose. After 10 to 14 days, depending on the cell line, colonies of GFP-labeled and mCherry-labeled cells were visualized in different fluorescence channels via a ChemiDoc Imaging System (Bio-Rad, California, USA). GFP-labeled colonies were imaged in the Alexa488 channel, whereas mCherry-labeled colonies were imaged in the Cy3 channel, resulting in two images of the cell culture flasks (Fig. 4A). The number of GFP- and mCherry-labeled colonies (> 50 cells) was counted with ImageJ software (version 1.53), and the OER was calculated as described for 2D radiosensitivity.

### Westernblot

Western blotting was performed as previously described [43]. Briefly, the cells were seeded in 6-well plates, lysed and homogenized, and the protein concentration was determined via the Bradford assay (Bio-Rad Laboratories, Inc., Hercules, CA, USA). The proteins were separated via SDS‒PAGE, transferred to PVDF membranes (Merck Millipore, Burlington, MA, USA), blocked, and then incubated with primary antibodies against HIF1α (1:1,000, BD Biosciences), CA IX (1:2,000, clone no. M75, Absolute Antibodies, Oxford, UK) and β-actin (1:5,000, Sigma‒Aldrich). HRP-conjugated secondary antibodies (polyclonal rabbit anti-mouse antibody and polyclonal goat anti-rabbit antibody, both DAKO Deutschland GmbH, 1:2,000) were used. An enhanced chemiluminescence (ECL) detection system (Clarity Western ECL Substrate or Clarity Max Western ECL Substrate, Bio-Rad) was used to visualize immune complexes with a ChemiDoc Imaging System (Bio-Rad). For quantification of protein bands, ImageJ software was used. The relative protein levels were determined as the quotient of the protein of interest and loading control (actin protein level).

### Immunohistochemistry (IHC)

IHC was performed as previously described [44]. For IHC staining, at least five spheroids per condition were fixed with 4% praformaldehyde at room temperature (RT) for 24 h. A 2% agarose solution of Type 1 A agarose (Merck, Darmstadt, Germany) or low-melt agarose (Carl Roth, Karlsruhe, Germany) was used for agarose embedment. To arrange multiple spheroids in one agarose block, a nylon 12 stamp with 66 pins was 3D printed (Xometry Europe GmbH) and coated with silicone. Prewarmed Type 1 A agarose was poured into a steel tray, and the stamp was pressed horizontally into the liquid agarose. At least five spheroids were pipetted into each well, followed by centrifugation at 300 rcf for 3 min at 4 °C. Liquid 2% low-melt agarose was then added until solidification.

The samples were dehydrated (Histocore PEARL, Leica, Nußloch, Germany), embedded in paraffin (Histocore Arcadia H, Leica) and sectioned (semiautomated microtome HM340E, Thermo Fischer Scientific) into 4 μm sections. The slides were then immersed sequentially and deparaffinized. For antigen retrieval, the samples were boiled in citrate buffer (pH = 6, Zytomed Systems, Bargteheide, Germany) for 50 min in a steam cooker. After retrieval, the slides were circled with an advanced PAP pen (Merck). Nonspecific binding was blocked with protein blocking solution (Antibody Diluent, Zytomed Systems) for 50 min. The slides were incubated with primary antibodies against CA IX (1:50, clone no. M75, Absolute Antibodies), HIF1α (1:100, BD Biosciences), Pimonidazole (1:200, Hypoxyprobe, Inc., Massachusetts, USA), GFP (1:200, Cell Signaling) and cleaved caspase-3 (1:150, Cell Signaling). After that, the slides were washed in washing buffer (Zytomed Systems), and secondary antibodies (anti-rabbit/mouse polymer HRP, DAKO) were added. Chromogenic detection was performed via a liquid DAB + substrate chromogen system (DAKO). Afterwards, the slides were counterstained with Mayer’s hemalum solution (1:5 dilution, Sigma‒Aldrich). Finally, the slides were dehydrated and then mounted with Eukitt (Sigma‒Aldrich). The dry slides were scanned on a slide scanner (Axio Sca Z1, Zeiss, Ostfildern, Germany).

**Fig. 1:**
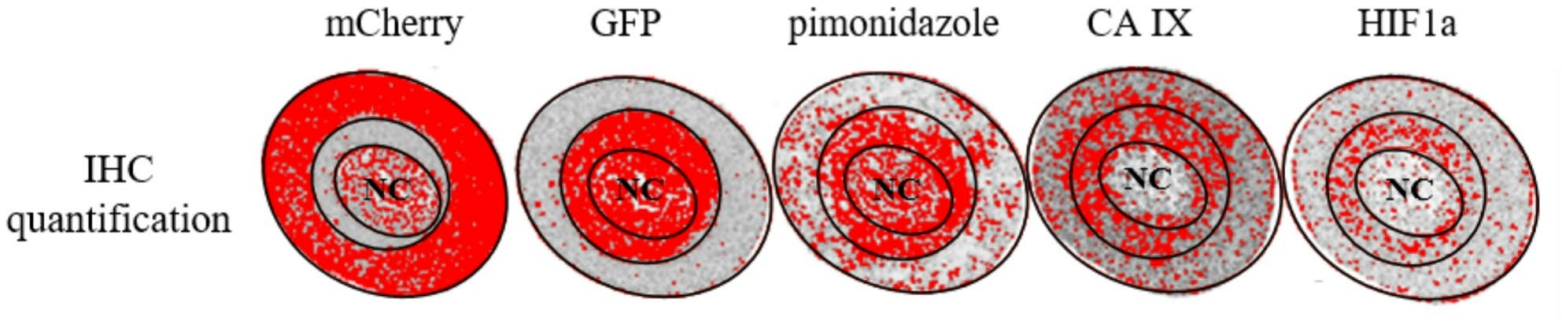
Shown are exemplary spheroid sections stained for mCherry, GFP, pimonidazole, CA IX, and HIF1α and their corresponding ImageJ-based quantification masks. For image analysis, spheroid sections were converted to RGB stacks, and DAB-positive areas (red overlay) were quantified separately within the GFP-positive inner layer and the mCherry-positive outer layer and the necrotic center (NC), which was excluded from the analysis. The resulting mean stained areas were used to calculate relative marker distribution between hypoxic and normoxic regions

For quantitative evaluation of mCherry, GFP, pimonidazole, CA IX, and HIF1α staining, ImageJ software was used (Fig. 1). Spheroid sections were converted into RGB stacks, and the blue channel was selected for analysis to optimize contrast of DAB-based immunostaining. Threshold levels were manually adjusted to ensure accurate detection of positive staining across all markers. Subsequently, mCherry- and GFP-positive regions were identified and used as spatial reference masks, onto which the pimonidazole-, CA IX-, and HIF1α-stained areas were overlaid. The mean stained area (in pixels) was quantified for the inner (GFP-positive) and outer (mCherry-positive) spheroid regions, and the relative ratios between both compartments were calculated to determine the spatial distribution of marker expression. The necrotic core was not included in the analyses, as it is irrelevant for the analysis of radiation sensitivity.

### mRNA sequencing and gene expression analysis

For mRNA sequencing, 2.5 µg of total RNA per sample was used. PolyA-based library preparation and sequencing were performed by Genewiz (Leipzig, Germany). Paired-end strand-specific sequencing was performed with approximately 2 × 20 million reads per sample via the Illumina NovaSeq platform. Adapter sequences as well as low-quality read ends were clipped off via Cutadapt (v 4.4) [45]. The processed sequencing reads were aligned to the human reference genome (UCSC hg38) via HISAT2 (v 2.2.1) [46]. SAMtools (v 1.19.2) was used to extract primary alignments and to index the resulting bam files. FeatureCounts (v 2.0.6) was used to summarize the gene-mapped reads [47, 48]. ENSEMBL (GRCh38 v110) was used as an annotation basis [49]. Differential gene expression was determined via the R package edgeR (v 4.2.1) via trimmed mean of M values (TMM) normalization and the exact test function [50, 51].

### Gene set enrichment analysis

Gene set enrichment analysis (GSEA) was performed via the R-packages clusterProfiler (v 4.12.1) and MSigDB gene sets (v2024.1. Hs) via the fgsea algorithm and setting the exponent parameter to 0 for unweighted analyses of log2-fold-change-sorted gene lists obtained from differential gene expression analyses [52, 53].

### Statistics

All data represent the mean value and standard deviation (+/±SD) of at least three independent experiments. The significance of differences was assessed via an unpaired two-tailed Student’s t test. A p value was considered to indicate a significant difference in reference to the indicated population of control cells. Significant p values are highlighted with asterisks (* *p* < 0.05, ** *p* < 0.01, *** *p* < 0.001)

## Results and discussion

### Effects of hypoxia in HNSCC monolayer cell models

**Fig. 2:**
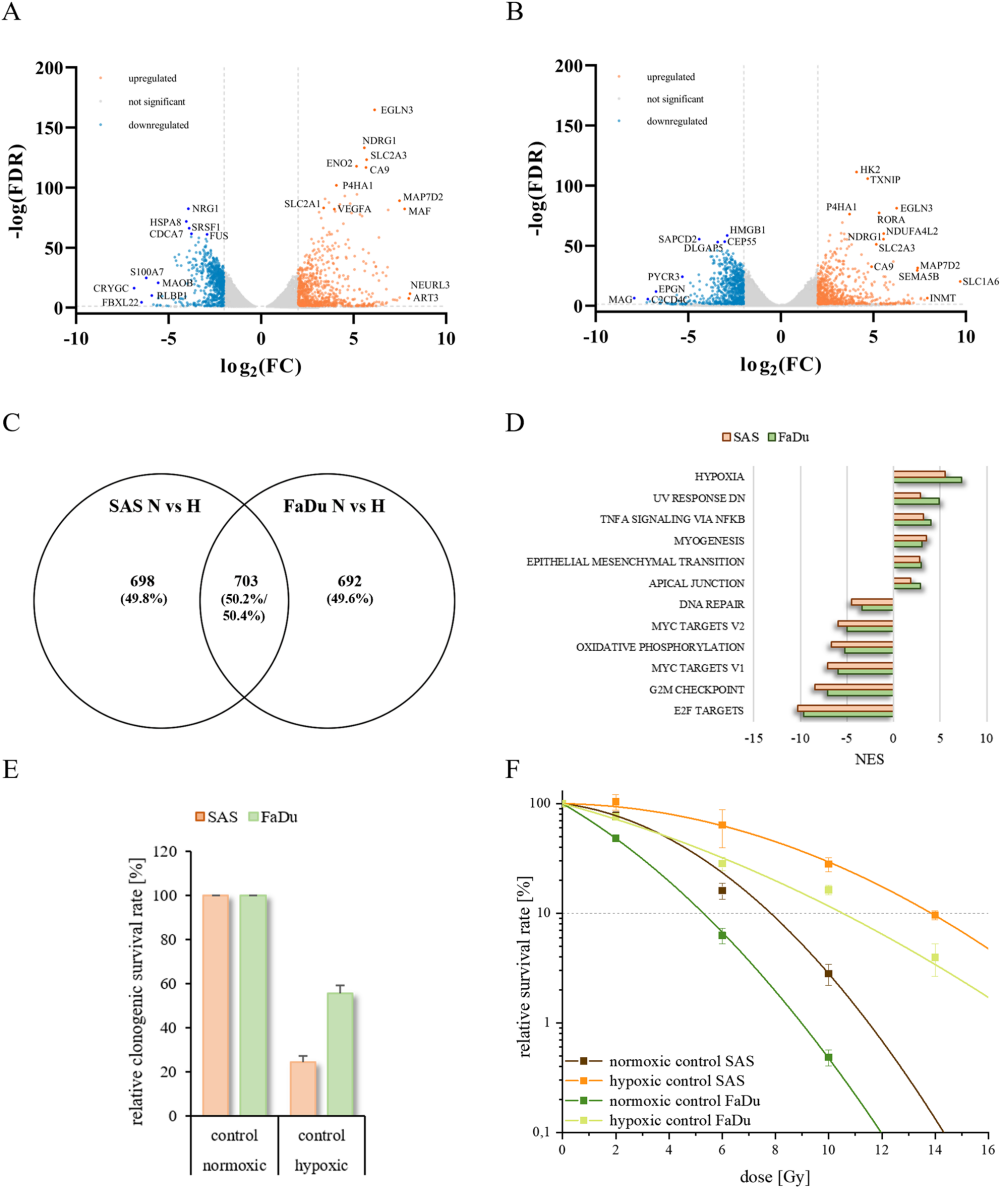
Gene expression analysis identified genes deregulated by hypoxia in both SAS and FaDu HNSCC. Volcano plot of differentially expressed genes in SAS (A) and FaDu (B) HNSCC under normoxic versus hypoxic conditions. Venn diagram (C) showing the overlap of differentially expressed genes from both transcriptome analyses filtered for significance of deregulation (FDR ≤ 0.05) and absolute expression level (log2(FC) ≥ 2) of the respective genes in SAS and FaDu cells [55]. Bar chart showing the top 12 significantly enriched/depleted MSigDB hallmark gene sets (highest/lowest NES (normalized enrichment score), lowest p-adjust) as determined by GSEA (D) on the basis of expression differences. Clonogenic survival (E) and radiosensitivity (F) of 2D HNSCC models under normoxic (21 % O2) and hypoxic (0.1 % O2) conditions. The diagrams (E/F) show the clonogenic survival rate [%] after irradiation for each HNSCC line, SAS (orange) and FaDu (green). The cells were irradiated with 2–14 Gy. The data represent the mean values (+/± SD) of at least three independent experiments. The dotted lines indicate a relative cell survival rate of 10 %. Significant p values are highlighted with asterisks (* p ≤ 0.05)

Two-dimensional (2D) cultures, are often used to determine radiation sensitivity of normoxic and hypoxic cultivated tumor cells. To compare the cell- and radiobiological behavior of hypoxic or normoxic 2D HNSCC cell lines SAS and FaDu we analyzed the RNA expression profiles, clonogenic survival and radiosensitivity. Both cell lines exhibited a similar hypoxic expression pattern (Fig. 2A and B). RNA-deep sequencing and gene set enrichment analysis revealed extensive hypoxia-associated transcriptional adaptation, with more than 1,300 differentially expressed genes per cell line, as expected, and strong enrichment of hallmark gene sets related to hypoxia (e.g. *CA9*,* P4HA1*,* EGLN3*,* NDRG1*,* SLC2A3*,* SLC1A6*) (Fig. 2C and D). Several hypoxia-regulated genes are modulators of radioresistance, highlighting the need for analyses that address hypoxic cell populations [54]. Clonogenic survival assays showed reduced survival and increased radioresistance of hypoxic compared to normoxic cultivated 2D HNSCC cell lines, with OERs of approximately 2.0 to 2.1 (Fig. 2E and F). These findings underscore the pronounced impact of oxygen availability on gene regulation, clonogenic survival and radiosensitivity in conventional 2D culture systems. To further translate these findings into a physiologically relevant 3D context, we established a two-color spheroid HNSCC model that enables spatial discrimination of hypoxic and normoxic cell populations in the same tumor model and their differential response to radiosensitivity.

### Establishment of two-color-HNSCC spheroids

#### Determination of the ratio of outer and inner cell layers of two-color HNSCC spheroids

**Fig. 3:**
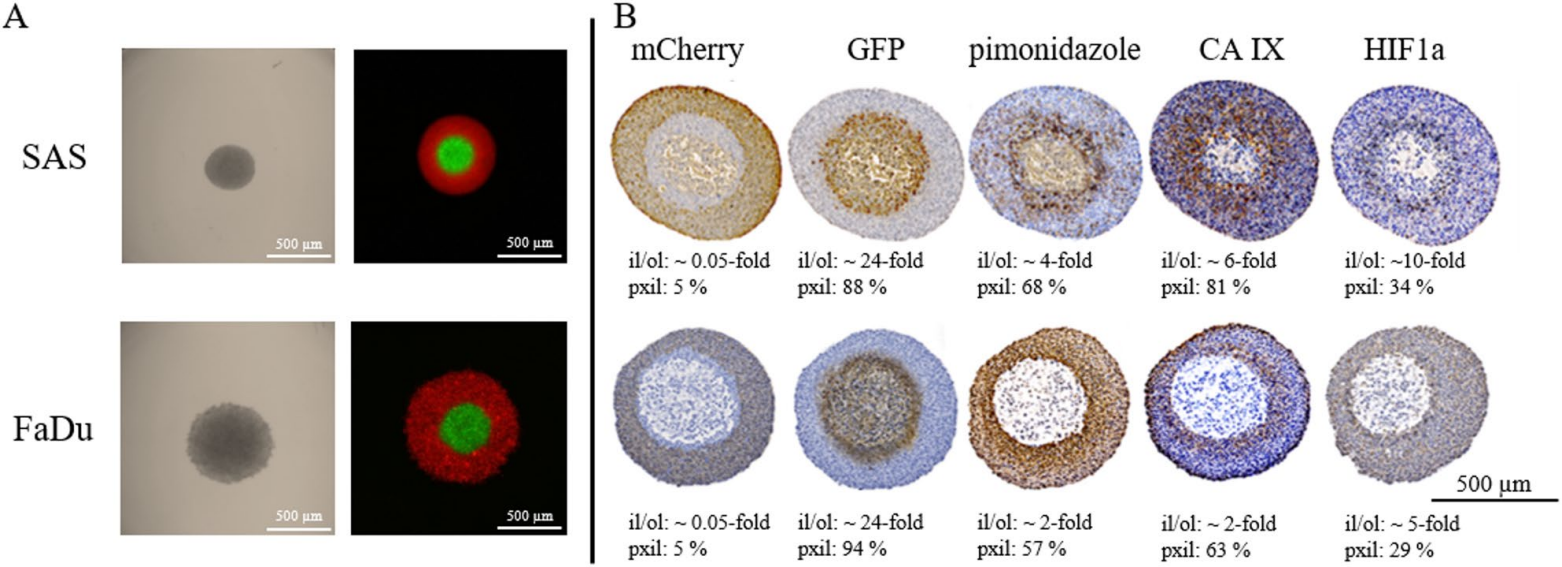
Fluorescence of vital two-color spheroids after 48 h (SAS, top) and 72 h (FaDu, bottom) (**A**) and histological staining (**B**) of mCherry, GFP, pimonidazole, CA IX and HIF1α of the corresponding fixed two-color SAS (top) and FaDu (bottom) spheroids. Quantification of positive staining (ol/il) in the respective layer (ol, outer mCherry-labeled layer and il, inner GFP-labeled-layer, pxil, positive pixels of the inner GFP-labeled-layer) is documented under each image. It should be noted that the necrotic core of the spheroid, which also showed partial IHC staining, was excluded from the investigations as it is irrelevant for the analysis of radiation sensitivity. Each image is representative of at least three independent experiments

To establish a two-color 3D model, two HNSCC cell lines (SAS and FaDu) were labeled with GFP and mCherry, respectively. The GFP-labeled cells represented the inner cell layer of the spheroids, whereas the mCherry-labeled cells represented the outer layer of the spheroids (Fig. 3A). To achieve separation of normoxic and hypoxic areas, a defined number of GFP-labeled cells were seeded first, followed by cells labeled with mCherry. At mixing ratios of 1:1 and 1:2, the mCherry-labeled cells are unable to envelop the GFP-labeled cells (Supplement Fig. 1). This is achieved from a ratio of 1:4. Additional IHC staining was conducted with the exogenous and endogenous hypoxia markers pimonidazole, CA IX and HIF1α to visualize hypoxic regions and confirming that the different labeled cells in the 3D spheroid model corresponded to hypoxic areas (Fig. 3). The GFP-positive inner cell layer (excluding the necrotic core) strongly correlated with pimonidazole, CA IX, and HIF1α staining at a ratio of 1:4 (Fig. 3B).The inner GFP-labeled cell layer shows an increase in the positive labelling of the hypoxia markers pimonidazole (SAS 4.0-fold, FaDu 2.0-fold), CA IX (SAS 6-fold, FaDu 2-fold), and HIF1α (SAS 10-fold, FaDu 4.2-fold), compared to the outer mCherry-labeled cell layer, in both HNSCC models (Fig. 3B). HIF1α exhibits the highest selectivity for hypoxia, but the lowest proportion of stained cells in the inner layer compared to pimonidazole and CA IX staining. Although the mCherry-labeled cells envelop the GFP-labeled cells at a ratio of 1:6, IHC staining with pimonidazole, CA IX and HIF1α shows a suboptimal spatial overlap of these markers with the GFP-labeled cells (Supplemental Fig. 1). The proportion of hypoxic cells, represented by pimonidazole, CA IX and HIF1α staining, in the outer mCherry-labeled cell layer increases 1.2- to 6.0-fold and decreased in the inner GFP-labeled cell layer to 0.5- to 0.9-fold in comparison to the 1:4 two-color spheroids (Supplemental Table 1). Overall, a mixing ratio of 1:4 between GFP- and mCherry-labeled HNSCC cells appears to be optimal for distinguishing between the outer normoxic and inner hypoxic cell layers in HNSCC spheroids.

The exogenous hypoxia marker pimonidazole showed a gradual intensity gradient from the inner to the outer spheroid region, while the endogenous hypoxia markers HIF1α and CA IX were strongly confined to the GFP-positive hypoxic inner cell layer (Fig. 3B). Furthermore, the partial colocalization of pimonidazole and endogenous hypoxia markers HIF1α and CA IX confirms that the used hypoxia markers are suitable for detecting hypoxic cell layers in this presented HNSCC spheroid model. This is also supported by colocalization analyses and clinical studies showing that pimonidazole and endogenous hypoxia markers such as HIF1α and CA IX exhibit at least partial colocalization in HNSCC [56–59]. Necrotic areas are indeed a characteristic feature of large 3D spheroids and reflect the physiological complexity of tumour tissue under diffusion-limited oxygen supply. Importantly, necrotic cells have no clonogenic potential and are therefore irrelevant for determining radiosensitivity and can be neglected [60].

After determining the optimal mixing ratio and verifying the inner GFP-labeled cell layer as a hypoxic region, the next step was to assess whether this layer and differed in their radiosensitivity from the outer mCherry-labeled cell layer. Therefore, clonogenic survival assays, considered the gold standard for evaluating radiosensitivity in vitro, were performed.

#### Clonogenic survival and radiosensitivity of two-color HNSCC spheroids under normoxia and hypoxia

Several 3D hypoxia-adapted systems have been developed, investigating growth, viability, and therapy effects under controlled oxygen conditions [21–24]. Multicolor “FUCCI” systems revealed the impact of radiation on cell-cycle distribution [27]. Genetic reporter approaches, such as 9×HRE-TK-GFP constructs or UnaG- and HIF1α-based lineage tracers, allow real-time or long-term visualization of hypoxic cell behavior and repopulation dynamics [25, 26, 28]. Likewise, microfluidic and multi-sample hypoxia chips enable precise oxygen modulation and high-throughput testing, substantially refining our understanding of microenvironmental gradients [29]. Unlabeled spheroid models relying on live/dead, PI, or γH2AX staining, as well as studies using HypoxiTRAK™, HIF1α, or pimonidazole, have provided complementary molecular or viability endpoints [30–32]. Most 3D tumor models focus on visualizing or quantifying hypoxia effects or on evaluating the clonogenic overall survival of the spheroid. More recent hypoxia-on-chip platforms, including naturally hypoxic jumbo spheroids and clinically relevant brachytherapy-on-a-chip systems, now reproduce physiologic oxygen gradients and spatially accurate radiation dose distributions in vitro, thereby extending the experimental possibilities of hypoxia research beyond conventional 3D assays [20, 34].

**Fig. 4:**
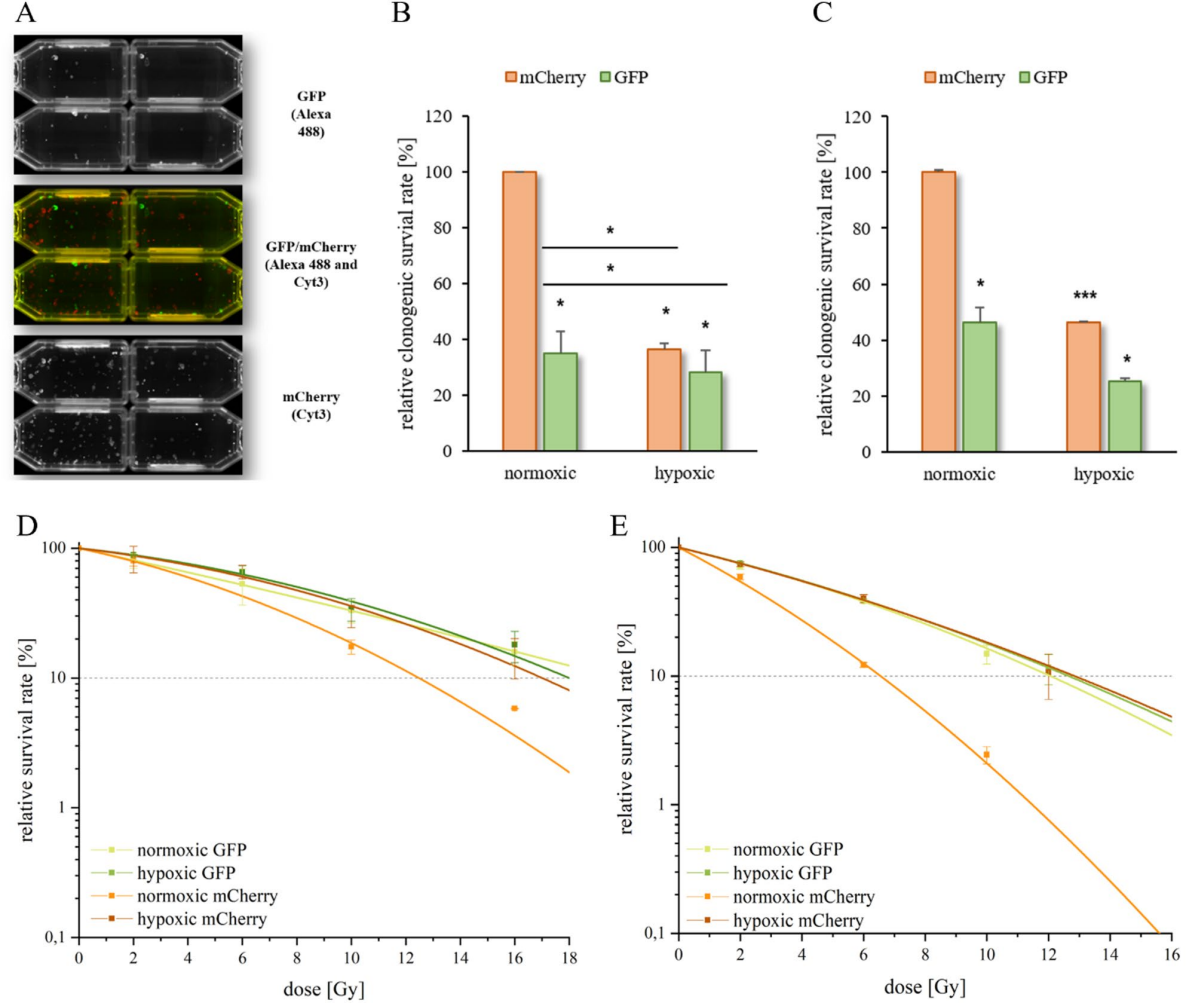
Two-color clonogenic survival of HNSCC cells under 3D cell culture conditions. HNSCC were incubated under normoxic (21% O_2_) and hypoxic (0.1% O_2_) conditions for 24 h. A two-color colony test (**A**) and the clonogenic survival rate [%] relative to the normoxic mCherry control cells for each HNSCC line, SAS (**B**) and FaDu (**C**), are shown. The diagram shows the radiosensitivity and survival curves of the different labeled SAS (**D**) and FaDu (**E**) cells; the value at 0 Gy was set as 100%. The spheroids were irradiated with 2–16 Gy. The data represent the mean values (+/±SD) of at least three independent experiments. Significant p values are highlighted with asterisks (*p ≤ 0.05, ***p ≤ 0.001)

Using the clonogenic assay, our two-colored HNSCC spheroid model enables the analysis of the clonogenic survival of individual layers through fluorescence-based visualization of the resulting colonies (Fig. 4A). The inner GFP-labeled spheroid layer showed markedly lower plating efficiency than the outer mCherry-labeled spheroid layer (Fig. 4B and C). Under normoxia, GFP-labeled cells reached only 35% (SAS) and 46% (FaDu) plating efficiency in comparison to the mCherry control. Under hypoxia, clonogenic survival further declined to 28% (GFP) and 36% (mCherry) in SAS, and to 25% (GFP) and 46% (mCherry) in FaDu spheroids, consistent with the known effects of hypoxia (Fig. 4B and C). The GFP-labeled cells of two-color spheroids irradiated under normoxic conditions (OER = 1.48–1.54) were similar in terms of radiosensitivity to the inner GFP- and outer mCherry-labeled cells of hypoxic cultivated spheroids (OER = 1.50–1.76) (Fig. 4D, E, Supplemental Table 2). Furthermore, the mCherry-labeled cells in the outer layer of the normoxic cultured spheroids were found to be the most radiosensitive (Fig. 4D, E, Supplemental Table 2). In contrast, radioresistance was detected in both cell layers (the outer mCherry-labeled and the inner GFP-labeled layer) if the 3D spheroids were cultivated under hypoxia (Supplemental Table 2). In summary, the cell colony formation assay, the gold standard for determining cell radiosensitivity, was utilized in this study to demonstrate that, in the presented two-layer 3D HNSCC model, the GFP-labeled cells of the inner hypoxic cell layer exhibited increased radiation resistance compared to the mCherry-labeled cells of the outer normoxic cell layer.

In contrast to our 2D cultures, which are frequently cultured in the Gas Pak EZ Anaerobic Pouch system, inducing anoxia to simulate oxygen deprivation, the presented 3D approach reproduces gradual oxygen conditions that reflect the in vivo tumor microenvironment. While 2D assays provide valuable information on hypoxia-induced radioresistance (OER = 2.0–2.1), they typically yield higher OERs than 3D systems (OER = 1.48–1.76), as they represent absolute rather than diffusion-limited anoxia. Consequently, 2D models fail to capture the spatially heterogeneous and dynamic nature of oxygen diffusion and consumption that governs cellular radiosensitivity in solid tumors [3]. Future experiments could include additional fluorescence-labeled cell layers to further resolve distinct microenvironmental zones within the spheroid, such as the necrotic core or hypoxic transition regions between inner and outer cell layers. Furthermore, the coupling of stable and hypoxia-induced fluorescent labelling could be further improved.

Taken together, our data demonstrated that the two-color spheroid HNSCC model thus enables the quantitative analysis of clonogenic survival within spatially defined hypoxic and normoxic compartments, which could form the basis for the screening of hypoxic radiosensitizers.

### Refinement of the two-color HNSCC spheroid model with ascorbic acid

HIF1α can be partially stabilized under normoxic conditions; to counteract this effect, application of ascorbic acid has been shown to promote HIF1α degradation [38]. This may be due to its antioxidant properties and ability to influence HIF1α-dependent signaling pathways [61–63]. As a cofactor of important hydroxylases, ascorbic acid regulates the level of HIF1α and thus affects the adaptation to hypoxia [31, 33, 34]. The effects of ascorbic acid on SAS and FaDu cells were assessed using the CellTiter-Glo assay, revealing no cytotoxicity (Supplemental Figs. 2 and 3) at a physiological concentration of 10 mg/l (0.06 mM), which was therefore applied in subsequent experiments [64]. In SAS and FaDu cells, ascorbic acid significantly reduced HIF1α protein levels (0.8-fold SAS and 0.5-fold FaDu) under normoxia, while hypoxia strongly induced HIF1α (1.6–2.0-fold) and CA IX protein level (60–200-fold) in both cell lines, independent of ascorbic acid treatment (Supplemental Fig. 4) [38]. Therefore, we further investigated the effects of ascorbic acid in the presented 3D HNSCC model.

#### Growth, density and protein expression levels of hypoxia and cell death markers in 3D HNSCC models

Treatment of the 3D HNSCC model with physiological concentrations of ascorbic acid (10 mg/l) slightly enhanced spheroid growth and density, resulting in mean diameters of 1125 ± 23 μm (SAS) and 1150 ± 35 μm (FaDu) compared with 1029 ± 19 μm (SAS) and 944 ± 28 μm (FaDu) in untreated controls (Supplemental Fig. 3). Similar effects were also observed in breast cancer cells [65].

**Fig. 5:**
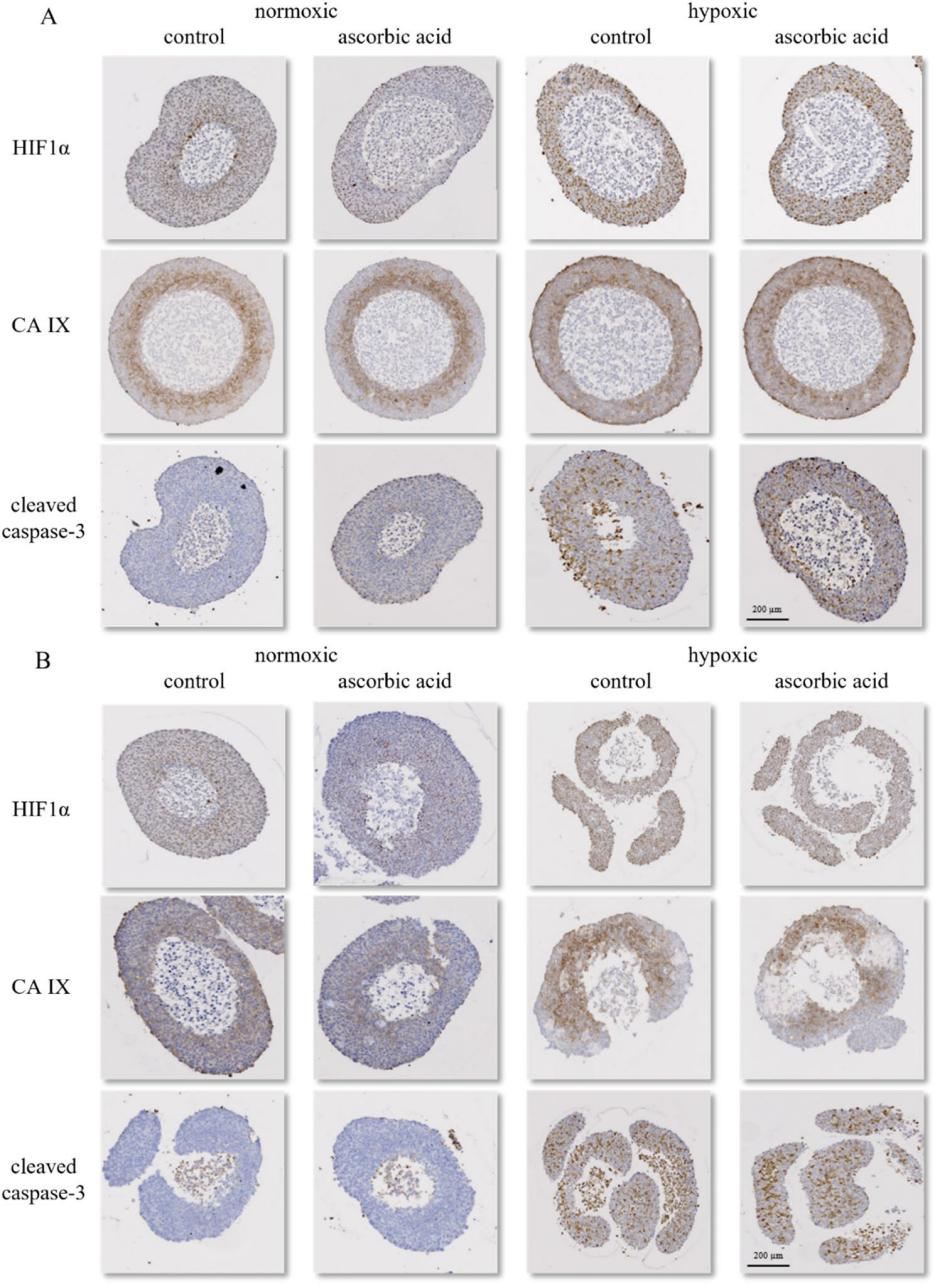
Histological staining of HIF1α, CA IX and cleaved caspase-3 in SAS (**A**) and FaDu (**B**) spheroids. The spheroids were cultured under normoxic and hypoxic conditions in media with and without physiological concentrations of ascorbic acid. The necrotic core was not included in the analyses. Each image is representative of at least three independent experiments

The protein expression of HIF1α, CA IX and cleaved caspase-3 with and without ascorbic acid treatment under normoxic and hypoxic conditions was examined in HNSCC spheroids (Fig. 5). The quantified IHC-data are shown in Supplemental Fig. 5. The levels of HIF1α (2.3–3.7 fold) and CA IX (1.4–2.3 fold) were elevated in the spheroids of both HNSCC cell lines cultivated under hypoxic conditions. Furthermore, under normoxia, the expression of the hypoxic markers HIF1α and CA IX is detectable in inner layers of the HNSCC spheroids as well, with lower expression after the ascorbic acid treatment (0.5–0.7 fold). The marker of apoptosis, cleaved caspase-3, was not detected in normoxic SAS spheroids, but in the inner layer of the normoxic FaDu spheroids. Hypoxic conditions increased cleaved caspase-3 levels (1.8–3.4 fold) in both SAS and FaDu spheroids (Fig. 5, Supplemental Fig. 5). However, ascorbic acid treatment did not influence cleaved caspase-3 protein level. Ascorbic acid is an essential cofactor for Fe²⁺/2-oxoglutarate-dependent prolyl hydroxylases, which hydroxylate HIF1α. This promotes its degradation under normoxic conditions. However, in hypoxia, limited oxygen availability and PHD inhibition by metabolic intermediates prevent this reaction, making ascorbate ineffective in facilitating HIF1α hydroxylation and degradation [37, 38, 66]. Consequently, our results show ascorbic acid’s regulatory effect on HIF stability is oxygen-dependent.

#### Clonogenic survival and radiosensitivity in 3D HNSCC models

**Fig. 6:**
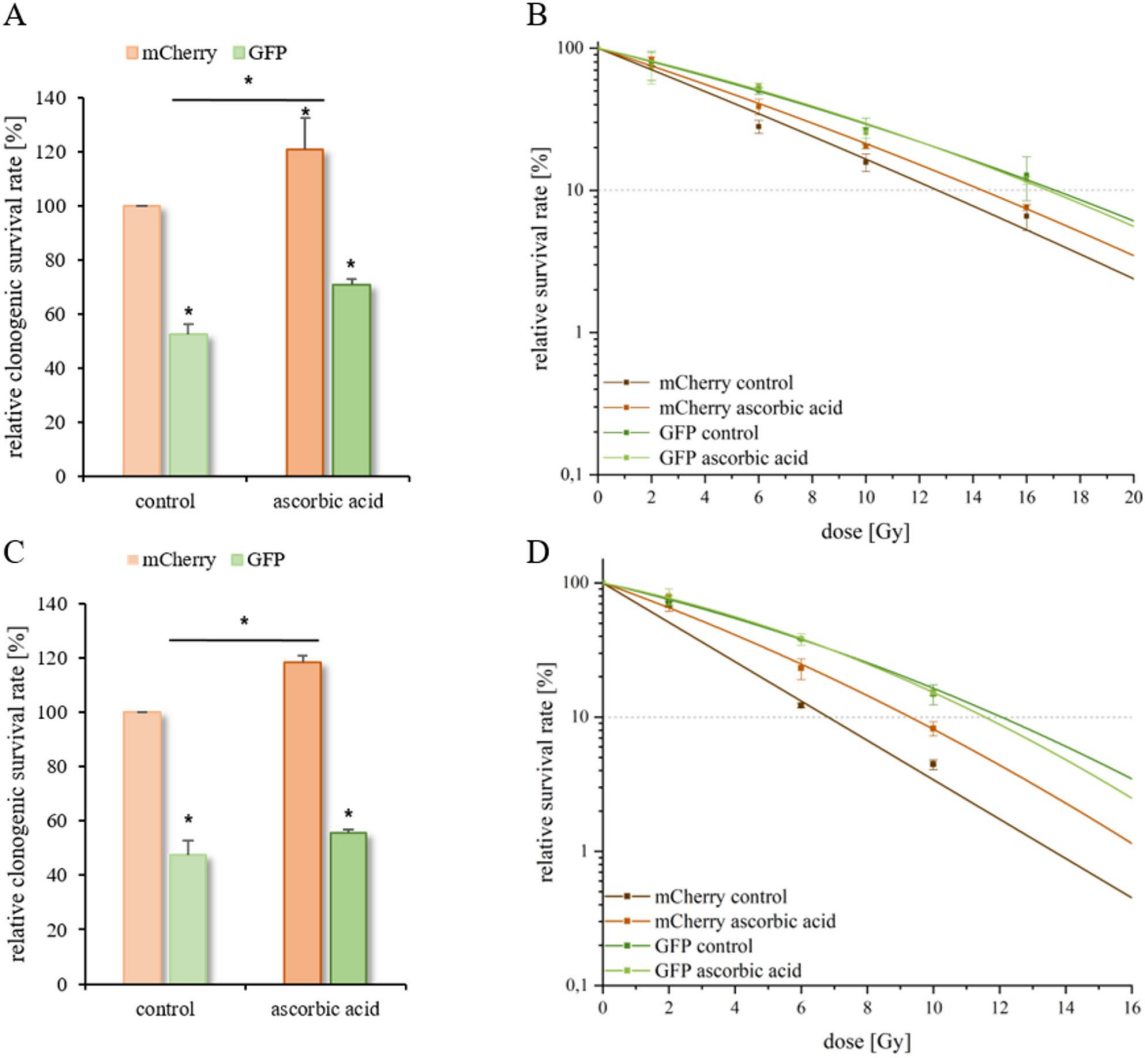
Clonogenic survival and radiosensitivity of mCherry- and GFP-labeled cells in a two-color spheroid model. HNSCC spheroids were incubated with ascorbic acid for 24 h. The diagrams show the clonogenic survival rate [%] relative to that of the mCherry-labeled control cells for each HNSCC line, SAS (**A**) and FaDu (**C**). The cells were irradiated with 2–16 Gy. The diagram shows the relative survival rates [%] and radiosensitivity of mCherry- and GFP-labeled SAS (**B**) and FaDu (**D**) spheroids treated with and without ascorbic acid. The data represent the mean values (+/± SDs) of at least three independent experiments. The dotted lines indicate a relative cell survival rate of 10%. Significant p values are highlighted with asterisks (*p ≤ 0.05)

To evaluate the effects of ascorbic acid we performed a clonogenic survival assay. Compared with that of untreated control spheroids, the application of ascorbic acid resulted in a slight increase in the clonogenic survival of mCherry- and GFP-labeled cells in both two-color spheroid HNSCC models (SAS and FaDu) by approximately 20% respectively (Fig. 6A and C). In addition, mCherry-labeled SAS and FaDu cells (outer layer) exhibited weak radioresistance (OER = 1.19–1.21) following ascorbic acid treatment in comparison with the untreated control cells (Supplemental Table 2). However, ascorbic acid had no effect on the radiosensitivity of the GFP-labeled cells (inner layer) in the SAS and FaDu spheroids (Fig. 6B, D). In agreement with the 3D data, the same effects for clonogenic cell survival and radiosensitivity were obtained in the 2D HNSCC models (Fig. 2 and Supplemental Table 2). Ascorbic acid exhibits a dual redox behavior, acting as an antioxidant and radioprotective agent at physiological levels, but as a potent pro-oxidant at pharmacological concentrations. High-dose ascorbate selectively increases intracellular hydrogen peroxide and lipid peroxidation in tumor cells with low catalase activity, thereby enhancing radiation-induced oxidative stress and clonogenic cell death while sparing normal cells [67, 68]. Nevertheless, the pharmacological effects of ascorbic acid require further investigation, since a radioprotective response was also observed in our two-layered 3D spheroid models.

Surprisingly, despite reduced HIF1α and CAIX expression in the mCherry-labeled outer layer, these cells displayed increased radioresistance after ascorbic acid treatment. This indicates that, under normoxic conditions, physiological concentrations of ascorbic acid acts as an antioxidant and radioprotective agent by scavenging radiation-induced ROS rather than serving as a pro-oxidant sensitizer [69]. Accordingly, the observed HIF1α suppression likely reflects metabolic stabilization rather than enhanced radiosensitivity, highlighting the dual redox role of ascorbic acid depending on oxygen availability and antioxidant capacity [70]. Radiobiological clonogenity is closely associated with the production of ROS [54, 71]. Ascorbic acid is a potent ROS scavenger and can therefore protect normoxic cells from radiation-induced oxidative damage [72–75]. However, these effects require further investigation in 3D models. In our study, ascorbic acid reduced HIF1α protein levels in both 2D and 3D systems, but exclusively in outer mCherry labeled layers of the spheroids. Moreover, ascorbic acid increased clonogenic survival in 3D cultures and resulted in radioresistance of the mCherry-labeled cell layer of the spheroid. The fact that similar effects could also be detected in our 2D models confirms the applicability of the two-colored 3D HNSCC model developed in the present study.

## Conclusion

The two-color 3D HNSCC model combined with a fluorescence clonogenic assay facilitates the distinction between hypoxic and normoxic regions within the same spheroids, thereby enabling a more detailed and functional analysis of normoxic and hypoxic cell layers in 3D HNSCC spheroids. These could be the basis for investigating hypoxic radiosensitizers. Furthermore, physiological supplementation of ascorbic acid reduces HIF1α levels of outer layers of the two-color HNSCC spheroids. Surprisingly the clonogenicity and radioresistance of HNSCC were enhanced after ascorbic acid treatment; these findings could be considered for further research. In future studies, this model should be tested in combination with other therapeutic agents. Subsequent studies are important to assess the applicability of the model in the context of combination therapies.

## Supplementary Information

Below is the link to the electronic supplementary material.

Supplementary Material 1

Supplementary Material 2

## Data Availability

The datasets used and/or analysed during the current study are available from the corresponding author on reasonable request.
