# Supplementary material for: Two-color spheroid model for determining the O2-induced radiosensitivity of HNSCC
Source: J Biol Eng. 2026 Jan 8;20:17. doi: 10.1186/s13036-025-00611-y (PMC12836854; doi:10.1186/s13036-025-00611-y)
Supplement: Supplementary file 1 — Supplementary Material 1 [file 13036_2025_611_MOESM1_ESM.docx]

# Supplement

# Supplemental Material 1


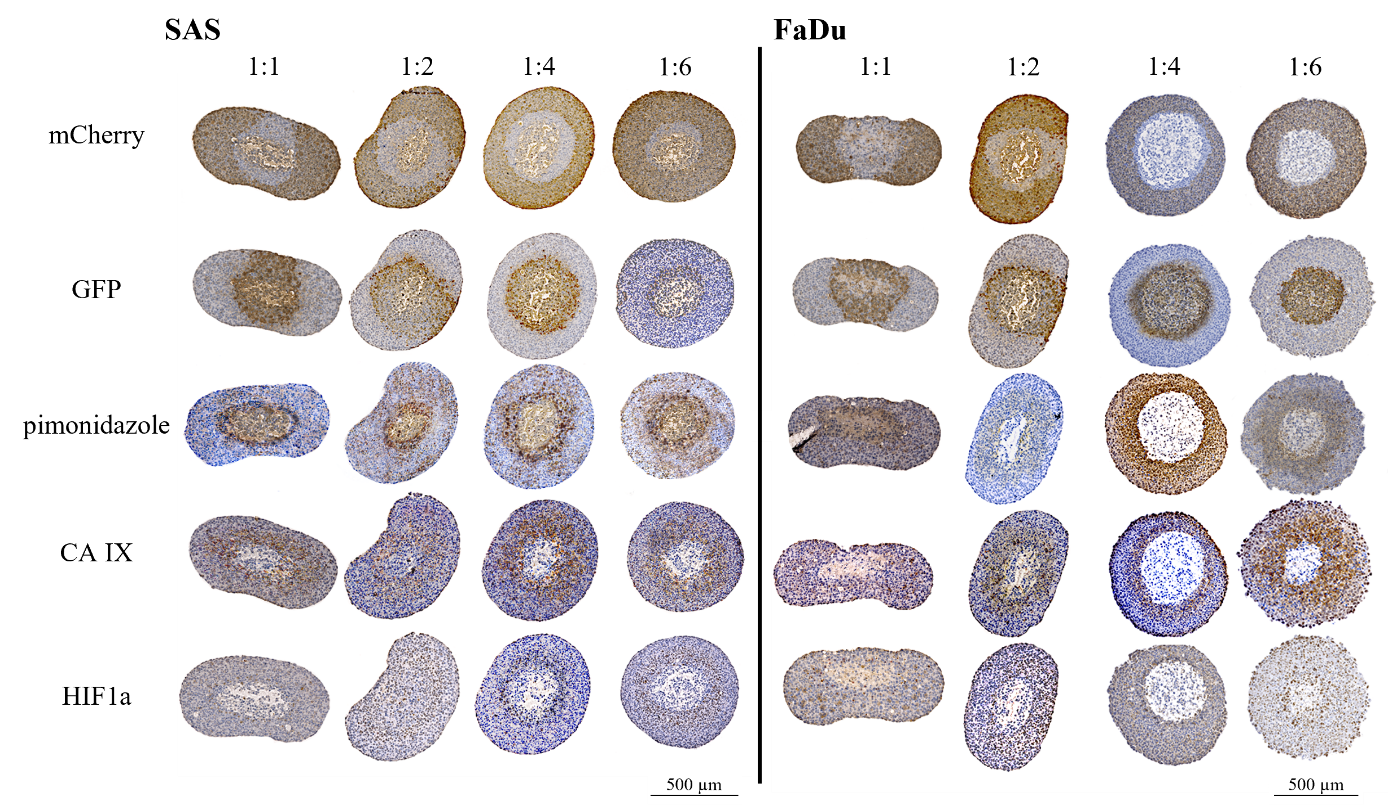


*Supplemental Figure 1: Seeding strategy of two-color SAS- (left) and FaDu- (right) spheroids and histological staining in four different mixing ratios (1:1, 1:2, 1:4 and 1:6) and the corresponding mCherry, GFP, pimonidazole, CA IX and HIF1α staining. Each image is representative of at least three independent experiments.*

Supplemental Table 1: The IHC staining`s of endogenous and exogenous hypoxia marker were quantified via ImageJ method for a ratio of 1:4 and 1:6 two-color SAS- and FaDu-spheroids. The relative positive IHC staining [%] is shown of the 1:6 ratio spheroid in comparison to the 1:4 spheroids. The relative positive IHC staining of the 1:4 spheroids was set to 100 %.

|  |  | SAS 1:6  [fold] | FaDu 1:6  [fold] |
| --- | --- | --- | --- |
| pimonidazole | **outer** | 1.98 | 1.20 |
|  | **inner** | 0.73 | 0.87 |
| CA IX | **outer** | 3.28 | 1.39 |
|  | **inner** | 0.61 | 0.82 |
| HIF1α | **outer** | 0.60 | 1.94 |
|  | **inner** | 0.47 | 0.78 |

# Supplemental Material 2

Supplemental Table 2: OER of monolayer cells and two-color spheroids relative to that of the normoxic or mCherry-labeled control. Significant p values are highlighted with asterisks (* p < 0.05; ** p < 0.01, *** p < 0.001).

|  | SAS |  | FaDu |  |
| --- | --- | --- | --- | --- |
|  | normoxic | hypoxic | normoxic | hypoxic |
| 2D control | 1.00 | 2.08 ± 0.15^**^ | 1.00 | 2.09 ± 0.15^**^ |
|  | mCherry | GFP | mCherry | GFP |
| 3D normoxia | 1.00 | 1.48 ± 0.15^***^ | 1.00 | 1.54 ± 0.04^*^ |
| 3D hypoxia | 1.50 ± 0.17^***^ | 1.64 ± 0.2^***^ | 1.58 ± 0.17^*^ | 1.76 ± 0.03^**^ |
|  |  |  |  |  |
| 3D | **SAS** |  | **FaDu** |  |
|  | mCherry | GFP | mCherry | GFP |
| control | 1.00 | 1.42 ± 0.08^***^ | 1.00 | 1.54 ± 0.04^***^ |
| ascorbic acid | 1.21 ± 0.09^**^ | 1.38 ± 0.08^***^ | 1.19± 0.10^*^ | 1.58 ± 0.11^***^ |

# Supplemental Material 3

## Cell cytotoxicity

To estimate the effect of ascorbic acid in 2D and 3D cell culture systems/models, the CellTiter-Glo® Luminescent Cell Viability Assay (Promega, Mannheim, Germany) was used in accordance with the manufacturer’s instructions. For determination of 2D systems, 2,000 SAS and 4,000 FaDu cells were seeded into 96-well plates (TPP, Trasadingen, Switzerland). For 3D cell culture systems, 50,000 SAS cells and 40,000 FaDu cells were seeded into coated 96-round-bottom-well plates (faCellitate).

In 2D systems, 24 h after plating, the cells were treated with different concentrations of ascorbic acid (10–10,000 µM) under normoxic and hypoxic conditions, whereas in 3D systems, the spheroids were treated 48 h (SAS) and 72 h (FaDu) after plating. Cell viability was measured 24 h and 96 h after treatment in the 2D and 3D HNSCC systems, respectively. The half-maximal inhibitory concentration (IC50) values were determined via dose‒response curve fitting via Origin 2019 (OriginLab Corp., Northampton, MA, USA).


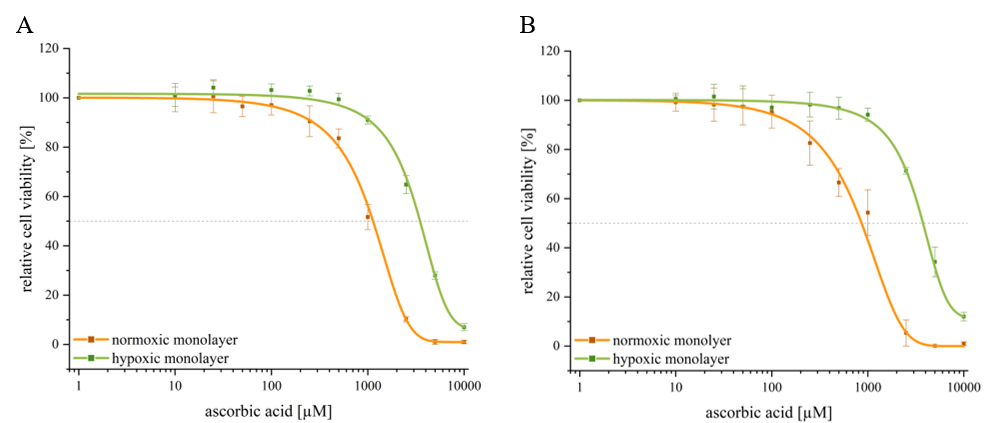
Supplemental Figure 2: 2D CellTiter-Glo Luminescent Viability Assay. Dose‒response curves of ascorbic acid treatment. The different HNSCC cell lines, SAS (A) and FaDu (B), were tested. The IC_50_ values of three independent experiments were determined from the fitted dose‒response curves. The dotted lines indicate a relative cell survival rate of 50%.


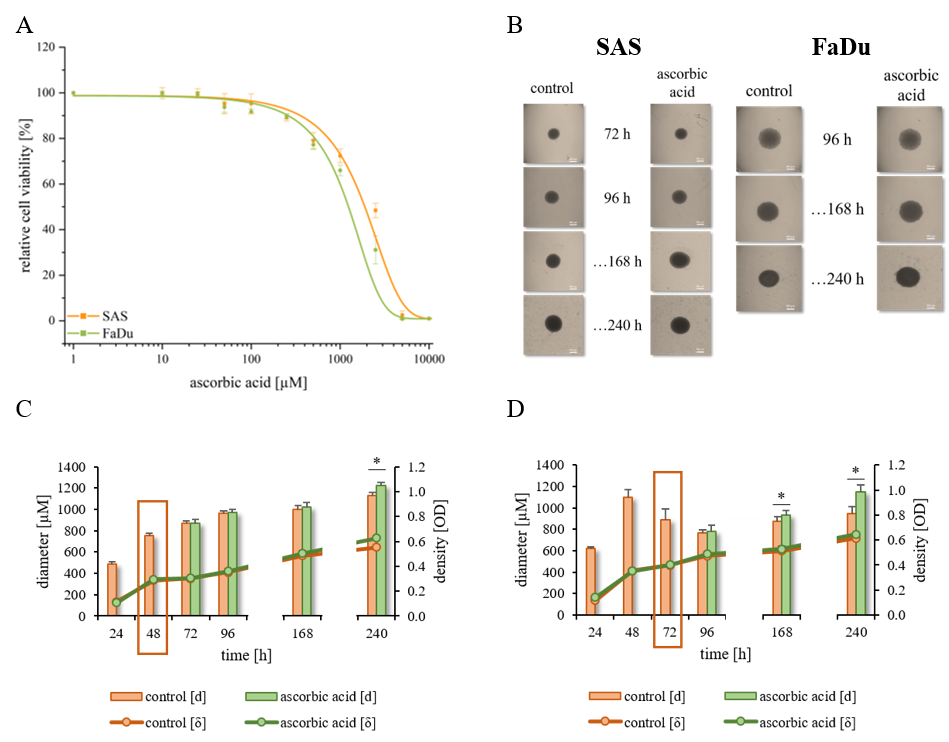
 Supplemental Figure 3: 3D CellTiter-Glo Luminescent Viability Assay (A). Dose‒response curves of ascorbic acid treatment. The different HNSCC cell lines SAS (orange) and FaDu (green) were tested. The spheroid growth of SAS and FaDu spheroids is shown in representative images (B). Influence of 10 mg/l ascorbic acid on spheroid growth and density 48 h (SAS) or 72 h (FaDu) after spheroid formation. The diameter (d, bars) and density (δ, lines) of the SAS (C) and FaDu (D) spheroids were analyzed with a Gelcount plate reader. The orange box indicates the start of ascorbic acid treatment. The data represent the average values (+/±SDs) of three independent experiments. Significant p values are highlighted with asterisks (* p < 0.05).

# Supplemental Material 4


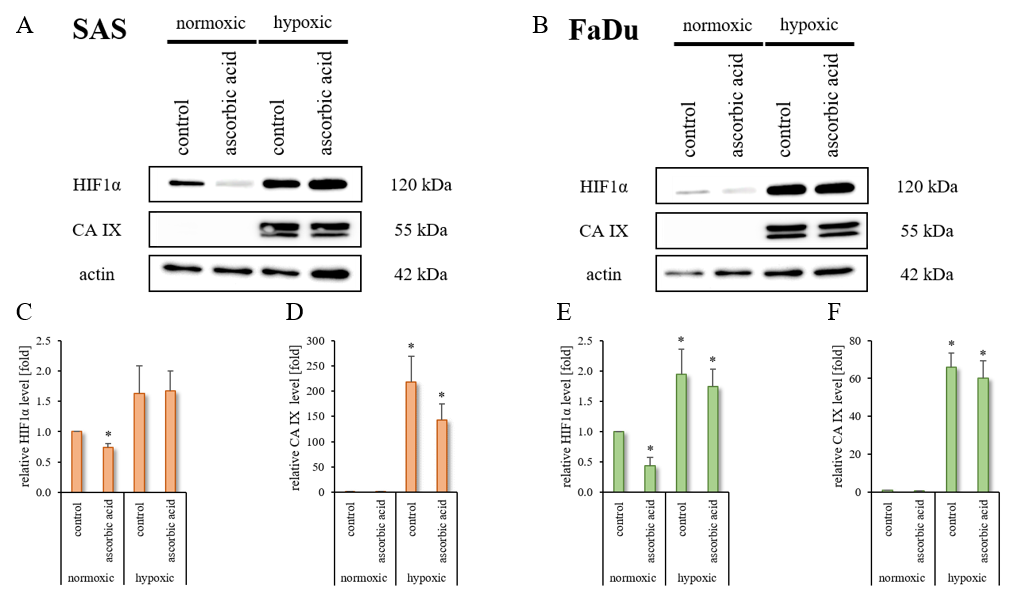


Supplemental Figure 4: Effects of hypoxia and ascorbic acid on HIF1α and CA IX protein expression levels in HNSCC cells. The SAS and FaDu cells were treated with ascorbic acid and cultured under normoxia or hypoxia. HIF1α and CA IX protein levels in SAS- and FaDu- cells were verified via Western blotting (A, B). The HIF1α and CA IX protein expression levels of SAS- (C, D) and FaDu- (E, F) cells were quantified. Actin was used as the loading control. The data (protein level) represents the mean values (+SD) of at least three independent experiments. Significant p values are highlighted with asterisks (* p ≤ 0.05).

# Supplemental Material 5


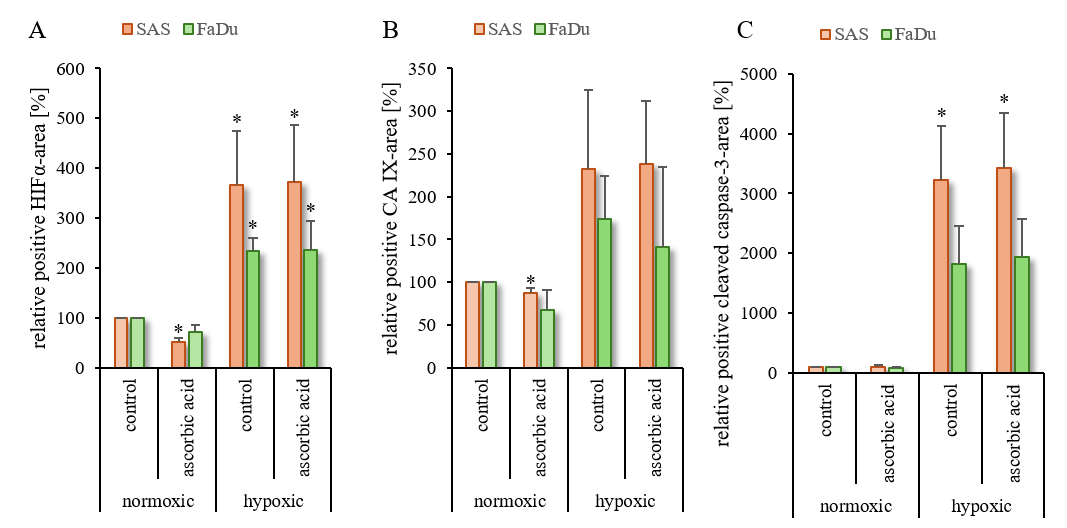


Supplemental Figure 5: Effects of hypoxia and ascorbic acid on HIF1α, CA IX and cleavaged caspase-3 protein levels in HNSCC Spheroids. The SAS and FaDu spheroids were treated with ascorbic acid and cultured under normoxia or hypoxia. HIF1α, CA IX and cleavaged caspase-3 positive areas in SAS- and FaDu- spheroids were verified via IHC (Figure 6), and quantified using Image J method (mean area detected). The data (relative positive area) represent the mean values (+SD) of at least three independent experiments. Significant p values are highlighted with asterisks (* p ≤ 0.05).
