# Supplementary material for: Two-color spheroid model for determining the O2-induced radiosensitivity of HNSCC
Source: J Biol Eng. 2026 Jan 8;20:17. doi: 10.1186/s13036-025-00611-y (PMC12836854; doi:10.1186/s13036-025-00611-y)
Supplement: Supplementary file 2 — Supplementary Material 2 [file 13036_2025_611_MOESM2_ESM.docx]

# Two-color spheroid model for determining the O_2_-induced radiosensitivity of HNSCC

Danny Knobloch-Sperlich^1*#^, Matthias Kappler^2*^, Markus Glaß^3^, Antje Güttler^1^, Marina Petrenko^1^, Jonas Pyko^4^, Tony Gutschner^4^, Frank Tavasol^2^, Dirk Vordermark^1^ and Matthias Bache^1^

^1^ Department of Radiotherapy, Martin Luther University Halle-Wittenberg, Ernst-Grube-Straße 40, Halle (Saale), Germany

^2^ Department of Oral and Maxillofacial Plastic Surgery, Martin Luther University Halle-Wittenberg, Ernst-Grube-Straße 40, Halle (Saale), Germany

^3^ Institute of Molecular Medicine, Section for Molecular Cell Biology, Faculty of Medicine, Martin Luther University Halle-Wittenberg, Halle (Saale), Germany

^4^ Institute of Molecular Medicine, Section for RNA Biology and Pathogenesis, Faculty of Medicine, Martin Luther University Halle-Wittenberg, Halle (Saale), Germany

* Both authors contributed equally to the manuscript.

# Correspondence: danny.knobloch-sperlich@uk-halle.de

**Supplementary Figures**


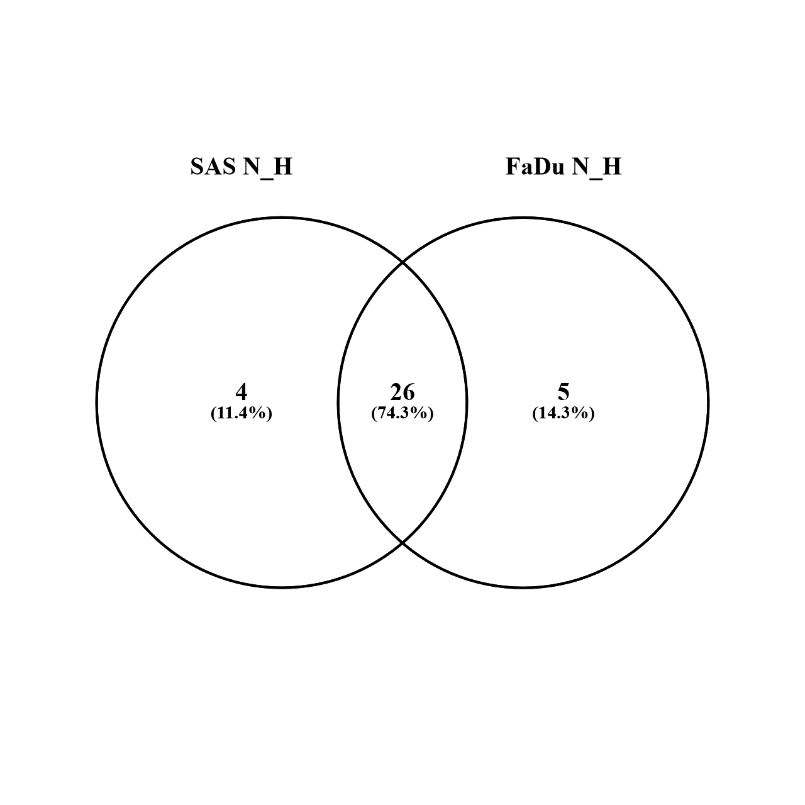


Supplemental Figure 1: VENN diagram of GSEA of mRNA-sequencing data considering all protein-coding genes. All HALLMARK Sets from SAS and FaDu cells were compared to each other [42].

| **A** | **B** |
| --- | --- |
| 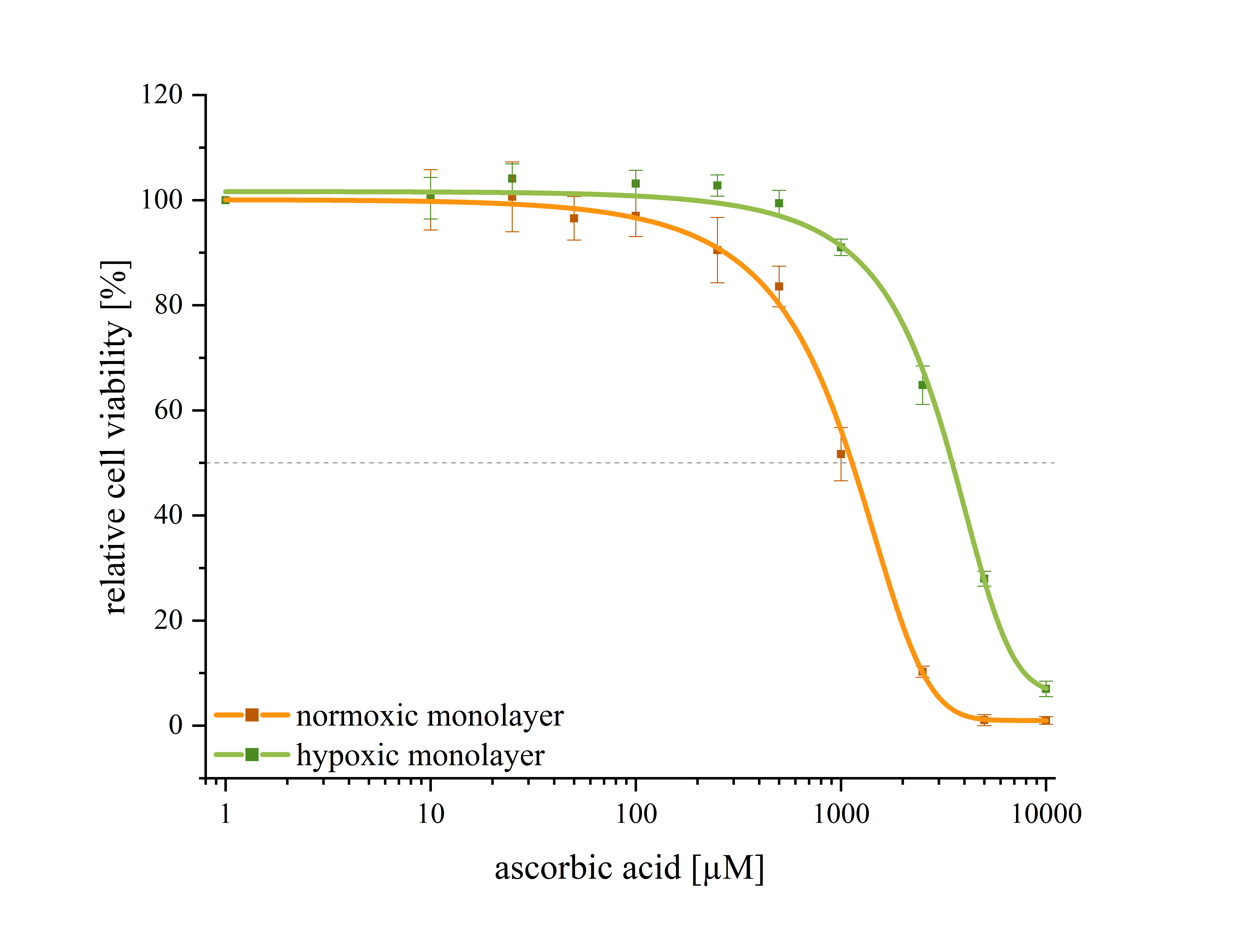 | 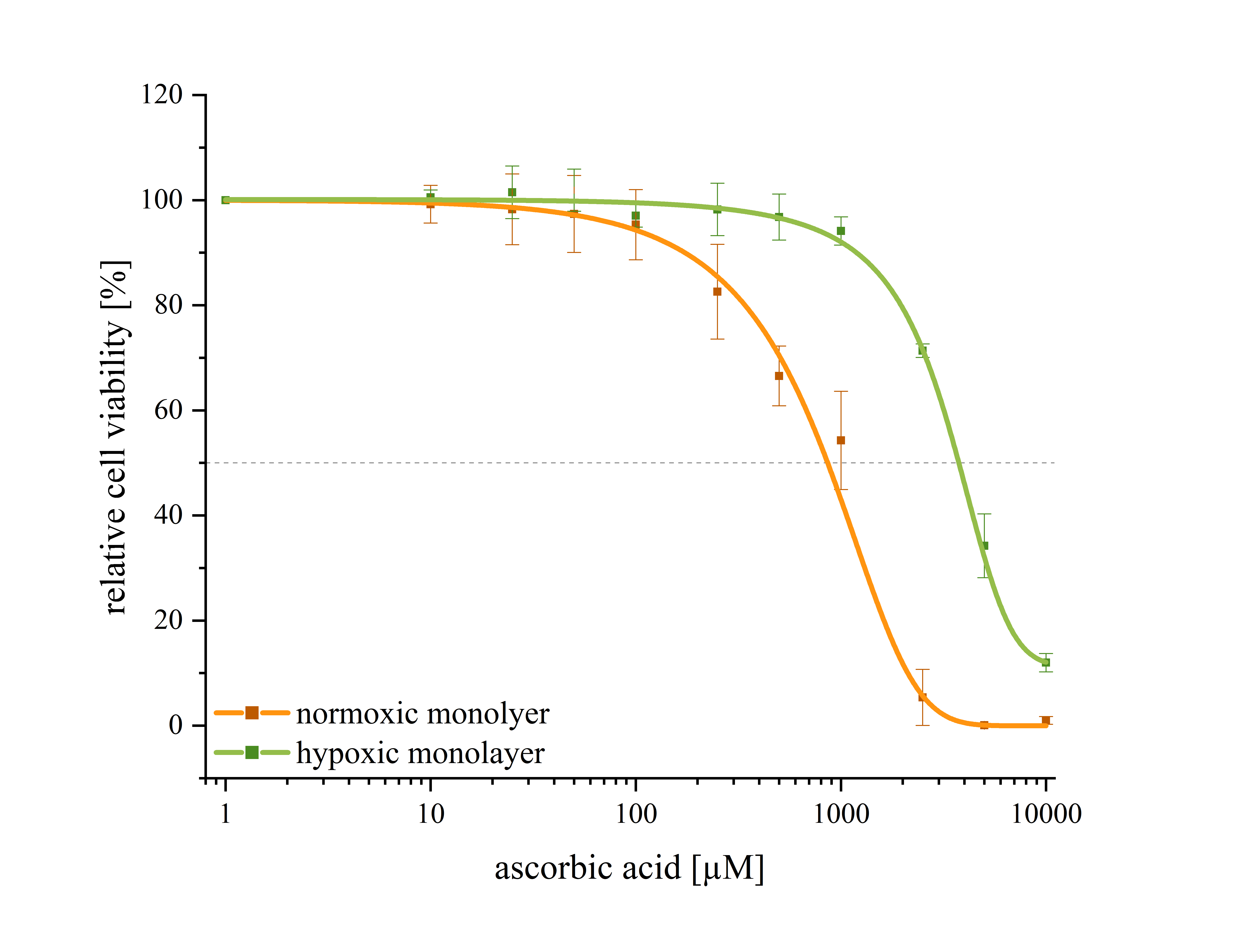 |

Supplemental Figure 2: 2D and 3D CellTiter-Glo Luminescent Viability Assay. Dose‒response curves of ascorbic acid treatment. The different HNSCC strains, SAS (A) and FaDu (B), were tested. The IC_50_ values of three independent experiments were determined from the fitted dose‒response curves. The dotted lines indicate a relative cell survival rate of 50%.

| **A** | 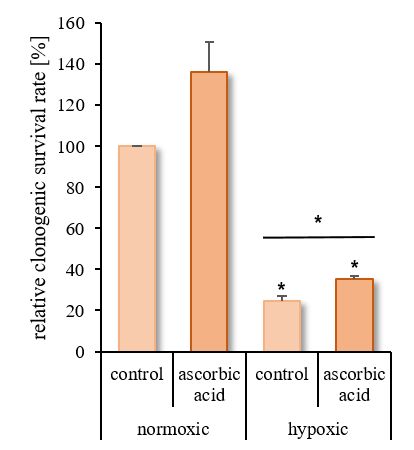 | **B** | 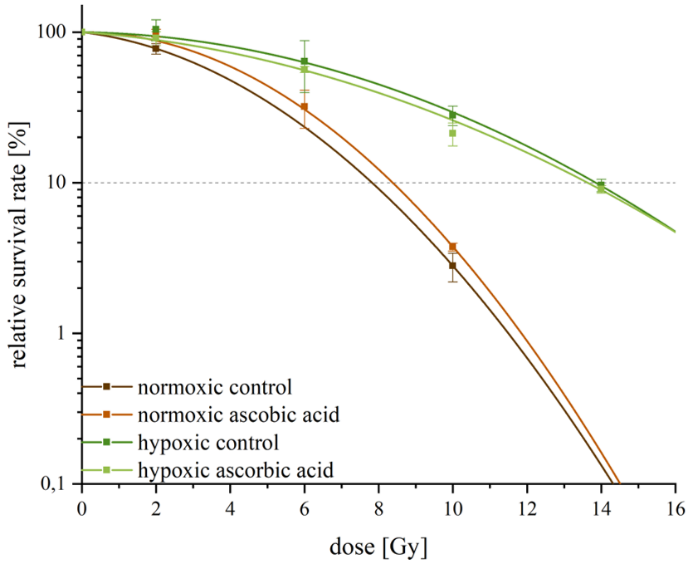 |
| --- | --- | --- | --- |
| **C** | 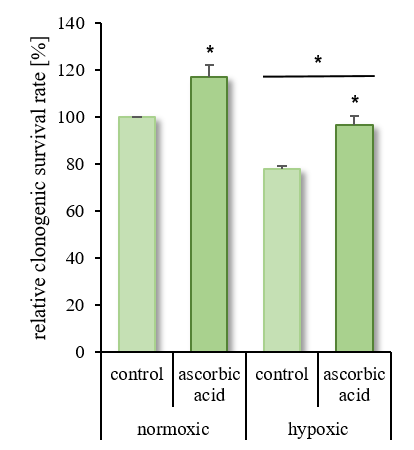 | **D** | 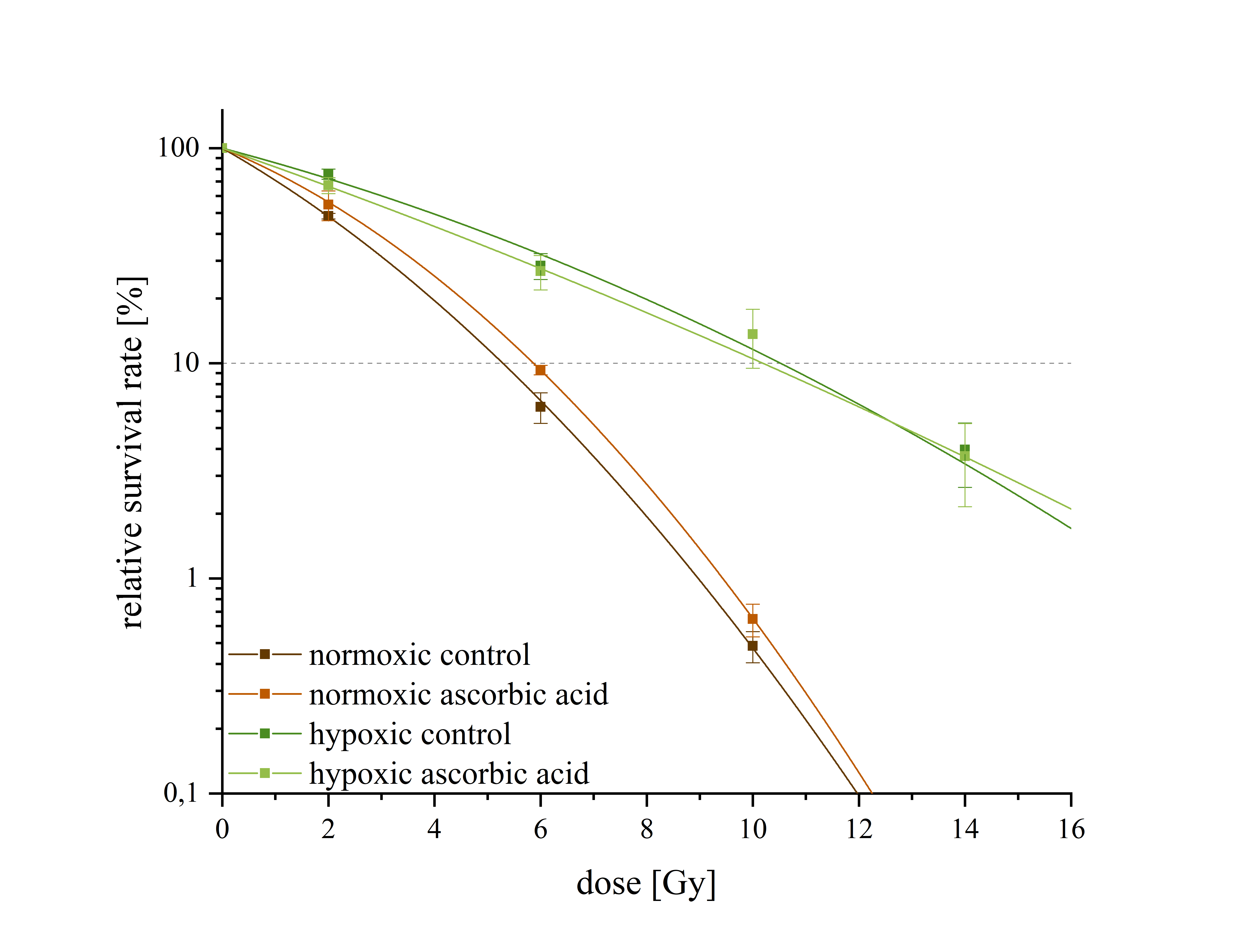 |

Supplemental Figure 3: Clonogenic survival and radiosensitivity of 2D HNSCC models with or without ascorbic acid under normoxic (21 % O_2_) and hypoxic (0.1 % O_2_) conditions. The diagrams show the clonogenic survival rate [%] without (A/C) or with (B/D) irradiation for each HNSCC line, SAS (A/B) and FaDu (C/D). The cells were irradiated with 2–14 Gy. The data represent the mean values (+/± SDs) of at least three independent experiments. The dotted lines indicate a relative cell survival rate of 10%. Significant p values are highlighted with asterisks (*p ≤ 0.05).

Supplemental Table 1: OER of monolayer cells and two-color spheroids relative to that of the normoxic or mCherry-labeled control. Significant p values are highlighted with asterisks (* p < 0.05; ** p < 0.01, *** p < 0.001).

| 2D | SAS |  | FaDu |  |
| --- | --- | --- | --- | --- |
|  | normoxic | hypoxic | normoxic | hypoxic |
| control | 1.00 | 2.08 ± 0.15^**^ | 1.00 | 2.09 ± 0.15^**^ |
| ascorbic acid | 1.34 ± 0.04^**^ | 2.04 ± 0.04^*^ | 1.12 ± 0.03^*^ | 2.05 ± 0.14^**^ |
|  |  |  |  |  |
| 3D | **SAS** |  | **FaDu** |  |
|  | mCherry | GFP | mCherry | GFP |
| control | 1.00 | 1.42 ± 0.08^***^ | 1.00 | 1.54 ± 0.04^***^ |
| ascorbic acid | 1.21 ± 0.09^**^ | 1.38 ± 0.08^***^ | 1.19± 0.10^*^ | 1.58 ± 0.11^***^ |
